# Mind the gap: scaling up the utilization of insecticide treated mosquito nets using a knowledge translation model in Isingiro district, rural south western Uganda

**DOI:** 10.1080/21642850.2020.1814782

**Published:** 2020-09-01

**Authors:** Ivan Mugisha Taremwa, Scholastic Ashaba, Carlrona Ayebazibwe, Imelda Kemeza, Harriet Ochokoru Adrama, Daniel Omoding, Jane Yatuha, Robert Hilliard

**Affiliations:** a Institute of Allied Health Sciences, Clarke International University, Kampala, Uganda; b Department of Psychiatry, Faculty of Medicine, Mbarara University of Science and Technology, Mbarara, Uganda; c Department of Information Technology, Uganda Christian University, Mukono, Uganda; d Department of Educational Foundations and Psychology, Mbarara University of Science and Technology, Mbarara, Uganda; e Infectious Disease Research Collaboration, Kampala, Uganda; f Department of Biology, Mbarara University of Science and Technology, Mbarara, Uganda; g Hospital for Sick Children, University of Toronto, Toronto, Canada

**Keywords:** Knowledge translation, insecticide treated mosquito nets, Uganda

## Abstract

**Background**: The phenomenon of Knowledge Translation (KT) is a key intervention towards bridging the ‘know–do’ gap. We conducted a KT initiative in Isingiro district to positively change attitude and improve on the uptake of Insecticide Treated Mosquito Nets (ITNs) as a malaria prevention strategy.

**Methods**: This was a community based interactive initiative that was carried out within the seventeen administrative units of Isingiro district using varied dissemination activities, namely: health talks; drama activities, and the sharing of ITNs success stories.

**Results**: We reached out to 34 dissemination groups, comprising communal gathering, religious crusades, open markets, secondary schools, and district administration. In addition, we spot-visited 46 households to ascertain the physical presence of ITNs, and their appropriate use. The major intervention was improved knowledge base of malaria causation and prevention strategies. The indicators for improved knowledge were hinged on the five-interventions, namely: (a) communal sensitization on malaria to provide, (b) monitoring and support of selected households, (c) emphasis of ITN use as a malaria prevention strategy, (d) promotion of care for ITNs, and (e) promotion of ITN use. In all, the major output was improved knowledge base of malaria causation and prevention strategies by providing accurate information to redress the myths and misconceptions related to malaria and ITNs use.

**Conclusion**: This undertaking describes a consolidated community intervention to promote ITN utilization. It is plausible that this intervention positively enhances and promotes uptake and utilization of ITNs.

## Introduction

Malaria is a major public health challenge whose burden and effects remain unacceptably high for a disease that can both be prevented and treated effectively (World Health Organization, 2017). This has necessitated scaling up the uptake of prevention measures to foster its control and elimination prospects (Roll Back Malaria Partnership, 2005). As such, increasing the coverage of insecticide treated mosquito nets (ITNs) has been a major focus to align with the World Health Organization (WHO) recommendations for malaria endemic settings. The malaria burden of Uganda remains high, with over 90% endemicity of the country's region (Ministry of Health, Uganda ). This has led to country-wide roll out of universal ITNs access strategy in which every two household members receive an ITN (Ministry of Health, Uganda ). Although this practice lead to a marked increase in household ITN possession, it has not always translated into their utilization and hence the full potential benefits have not been realized (Nyavor et al., 2017; Pulford, Hetzel, Bryant, Siba, & Mueller, 2011; Taremwa et al., 2017). Consequently, the phenomenon of ITN possession, but non-use urgently requires evidence based health promotion to bridge the ‘know–do’ gap, to elucidate the aptness of an intervention and upscale uptake (Buse & Hawkes, 2015). One such approach to achieve this is through health literacy using a knowledge translation (KT) model (Canadian Institutes of Health Research. Canadian Institutes of Health Research. Ottawa: Canadian Institutes of Health Research, 2004; Henderson, 2011; LaRocca, Yost, Dobbins, Ciliska, & Butt, 2012). Health literacy refers to implied achievement of knowledge level, skills and confidence to uptake actions that alleviate individual and community lifestyles and health (LaRocca et al., 2012; Ownby, Acevedo, Waldrop-Valverde, Jacobs, & Caballero, 2014). This approach has long been considered an important traditional approach in the implementation of community-based malaria prevention and control in sub-Saharan Africa (SSA) through media coverage like radio talk shows, television, brochures, and more recently, communities’ participatory techniques (Ownby et al., 2014).

The paradigm of KT is an emerging approach to harness the power of scientific evidence through a multi-stakeholder and multidisciplinary approach involving the research teams and the communities to collaboratively disseminate and apply research findings to offer timely solutions and transform practice (Canadian Institutes of Health Research. Canadian Institutes of Health Research. Ottawa: Canadian Institutes of Health Research, 2004; Henderson, 2011; LaRocca et al., 2012). Further, KT is regarded as the synthesis, exchange, and ethically-sound application of research knowledge within a complex system of interactions among research teams and communities to enhance the community-integration in the intervention for better health outcomes (Henderson, 2011; LaRocca et al., 2012). Premised on this, KT aims at accelerating the benefits of research through improved health, more effective services, and a strengthened healthcare system (Henderson, 2011). To achieve these, various KT models have been explored. These are: (a) the Canadian Institutes of Health Research (CIHR) model that is based on a cyclic conceptual guide for the overall KT process (Canadian Institutes of Health Research. Canadian Institutes of Health Research. Ottawa: Canadian Institutes of Health Research, 2004). It involves KT1 to define the research questions and methodologies, KT2 to conduct participatory research, KT3 to disseminate research findings, KT4 to place such findings in the context of other knowledge and sociocultural norms, KT5 that guides decision making and actions informed by research findings, and KT6 that influences subsequent research activities based on the impact of knowledge use (Canadian Institutes of Health Research. Canadian Institutes of Health Research. Ottawa: Canadian Institutes of Health Research, 2004). Besides CHIR, other KT models exists, such as: (b) interaction-focused frame work which provides practical guidelines by research teams to engage in the KT process and increase their familiarity with and understanding of the intended communities (Jacobson, Butterill, & Goering, 2003). Also, (c) context-focus modeling and frameworks that are used to understand the contextual factors that play important roles in the success or failure of the KT effort (Logan, Harrison, Graham, Dunn, & Bissonnette, 1999). (d) The knowledge-to-action (KTA) framework that facilitates the use of research knowledge by several stakeholders, for example practitioners, policymakers, patients, and the public (Graham et al., 2006). The KTA framework is hinged on two components, namely: knowledge creation, and the action. This approach emphasizes the collaboration between the research teams and communities (Field et al., 2014; Graham et al., 2006).

Our understanding of the ITN use as a nightly malaria prevention is based on a previous field survey that assessed the knowledge, attitude and behavioral practices towards the use of ITNs in Isingiro district (Taremwa et al., 2017). From that study, numerous barriers to ITN use were identified; notably the deficit in malaria awareness whose associated signs and symptoms are non-specific and often considered for pregnancy and other illnesses. To address these, we developed a multi-pronged approach to improve ITN uptake, with emphasis on targeted end user directed health education on the positive attributes of ITNs to make the behavior more socially normative and promote resiliency of ITN use. To support this, we went to the communities of Isingiro district with a field based KT program based on the collaborative approach to community-based malaria prevention aimed at positively changing attitude, and improving ITN uptake as a nightly malaria prevention strategy.

## Methods

### Study area, setting and duration

Isingiro district (0.84° S, 30.80° E) is located in southwestern Uganda, about 297 Kilometers from the capital city – Kampala, and 47 Km from Mbarara town. The district has a population of 486,360 based on projections from the 2014 census (Uganda Bureau of Statistics 2016, The National Population and Housing Census, 2014), and seventeen administrative units (15 sub-counties and 2 town councils) as indicated in Table 1. The district experiences tropical savannah climate with an average annual rainfall of 1200mm, and temperature range of 17–30°C. The district has two rainy seasons in each year: March to April, and September to November. According to Isingiro district-five-year district local government development plan II 2015/2016–2019/2020, the malaria positivity rate in 2014/2015 was reported at 38.2% (Isingiro district local government five year district local government development plan II 2015/2016-2019/2020). Additionally, evidence from the Ministry of Health Uganda indicates an upsurge of malaria infection in Isingiro distict (Ministry of Health, Uganda). It has a proportionally fair ITN coverage, estimated at 84.0% (Taremwa et al., 2017).

**Table 1.** Showing the respective village, parish and sub-county covered.

Village	Parish	Sub County
Rutsya	South	Kaberebere T/C
Kyenyenji	East	Kaberebere T/C
Rwemango	Kabugu	Kabuyanda S/C
Kitoma I	Kyera	Birere
Nyakakoni.C.	Nyakanoni	Masha
Rwenshebashebe	Rukuuba	Masha
Mpoma I	Ruborogota	Ruborogota
Kagamba	Kyarujagu	Kabingo
Endiinzi Town Board	Endiinzi Town Board	Endiinzi
Kaswina.B	Kashojwa	Ruggaaga
Kigarama	Iryango Ward	Kabuyanda T/C
Rwemizo	Rwamwijuka	Kikagate
Kashaasha	Mirambiro	Rushaasha
Karundi I	Ruhiira	Nyakitunda
Nyakibare. I	Nyamuyanja	Nyamuyanja
Kigando	Kyabishaho	Isingiro Town Council
Katahooka	Kyabahezi	Mbaare
Ntenga. B	Kigaragara	Kashumba
Kabengo. I	Burungamo	Ngarama

This study was guided by the identified gaps of the previous research that sought to assess the knowledge, attitude and behavioral practices of ITNs (Taremwa et al., 2017). In a unit, a parish was selected, and mobilization was done through local, and religious leaders. This intervention was carried out during the months of April to July, 2019.

### Strategies used

This community based dissemination exercise used a mixed method approach. The team held community based dissemination activities, and a meeting with Isingiro district administration. The discussion with district authorities aimed at improved practice in regard to the quality and quantity of ITN distribution. To the community, we used gatherings in the places of worship (churches, mosques), commercial centers, schools, and parish meetings. We identified volunteers with ITN success stories, and emphasized correct and consistent ITN use. This intervention targeted male and female adult (above 18 years) participants who were able to give informed consent.

### KT modeling and activities

Informed by a previous research study (Taremwa et al., 2017), use of ITNs in Isingiro district was below the expectation. This is supported by the fact that while 84.0% of the surveyed households possessed ITNs, only 66.1% consistently utilized them (Taremwa et al., 2017). There were numerous barriers that hindered their use, and these compounded the interventions of this KT approach. The barriers to ITN utilization were as a result of three factors, namely: (a) false beliefs and concerns about adverse risk of chemical in the ITNs, (b) ITNs of poor size and (c) lack of access to ITN. To efficiently address these, targeted multi-pronged interventions in the form of directed health education to emphasize the positive attributes of ITNs towards malaria prevention have been considered in this study. Also, a tell of success stories on the benefits of ITN utilization makes the uptake of ITNs more socially normative and promotes resiliency of use. Our community dissemination activities were based on KTA and interaction-focused KT models. These approaches were preferred as they ensured more engagement of research teams and communities (Graham et al., 2006; Ownby et al., 2014). As a result, there is a firm and synergistic bonding of the interaction (Field et al., 2014; Ownby et al., 2014).

The interventions were: (1) communal sensitization meetings and health talks on malaria infection, its presentation and prevention, (2) monitoring and support through random door-to-door home visits, (3) emphasis of ITN utilization as a malaria prevention strategy, (4) emphasis regarding the care for ITNs, and (5) promotion of the ITN use through community based initiatives.

The interventions, and their respective descriptions were:

- Communal sensitization on malaria; this was used for advocacy and sensitization led by members of the team and a VHT. It was the core intervention in all the 19 villages selected from the 17 administrative units of Isingiro district. The intervention followed an earlier report by the District Health Officer (DHO) that malaria has partly remained due to non-committal use of ITNs. In his narrative, it emerged that: *‘Even if people have bed nets (ITNs), they seem not to be using them. … . it is likely that they (communities) are not effectively putting ITNs to use, unless they believe it is an effective tool to protect them from malaria. For that, they need to understand the connection between mosquitoes and malaria.’ (District Health Officer).* We provided clear and accurate information to redress the myths and misconceptions related to malaria and ITNs. This was done through communal health talks, songs in the local language, plays, short drama performances composed of two people arguing about the causes of malaria and the third person would come in to join the debate to clarify the actual causes before the scenario was opened to the audience; poems and malaria brochures.
- Monitoring and support**:** through spot-checks of a few households, the presence of ITNs was confirmed and that they were hung properly, and their physical condition was adequate to be functional. Forty six households from six sub-counties (Kabingo, Rushaasha, Kashumba, Nyamuyanja, Birere, and Kabuyanda T/C) were randomly selected.
- Use of ITN as a malaria prevention strategy: to change behavior and social norms, the team emphasized that use of ITNs is one of the most effective methods of preventing malaria. We emphasized the need to keep the ITNs clean and ensure their proper use by hanging them up during the day and repairing any damages quickly.
- Care for the ITNs: the team re-echoed the message of good care for the ITNs. Accordingly, the community still had a challenge of the nets getting dirty either due to muddy houses, or smoke from the candle used as the source of light.
- Promoting ITN use: the volunteers shared different success stories to emphasis the positive attributes of ITN use.

To achieve these interventions, the following activities were conducted for the KT dissemination; (a) organized health talks within communities; (b) dissemination through a drama group who performed health education songs and a play about the causes, effect, signs of malaria and malaria prevention using ITN; (c) information, education and communication (IEC) using malaria brochures; (d) formation of women groups to champion ITN utilization strategy; (e) formation of school-based malaria prevention clubs; (f) organization of groups to continue the dissemination activity; and (g) the sharing of ITN success stories. The health talk was conducted in the local language (Runyankore), and it was supplemented with other dissemination activities. Prior to community entry, the study team and the Village Health Teams (VHTs) who supported KT prepared adequately as these were experienced health professionals who had actively been involved in community research. These teams carried out rehearsals related to the dissemination activities at least two-days before the scheduled event. The VHTs are community volunteers with health focus to mobilize and sensitize communities for active participation and utilization of health services. The VHTs work as an entry link to communities and health facilities. Further, the role plays were rehearsed by the drama group prior to the field intervention activities.

### Data extraction and analysis

Data were collected based on the dissemination activities using observation and unstructured interviews. Data collection was done by the team leader assisted by four members of the team who are proficient in the local languages (Runyankore) in the district. While in the community, there was extensive debriefing of study staff, which entailed the discussion of the emerging themes in observations and interviews. The conduct of dissemination and intervention activities (that is; focused health talks, formation of women volunteer groups, formation of malaria prevention clubs, organization of VHT groups and sharing of success stories) included informal conversations with the communities and also allowed the research team to observe the practices related to ITN use. These were recorded as hand-written field notes in either the local language or English, depending on the preference of the field staff and subsequently typed up (and translated if necessary) in English. The qualitative data collection approach enabled triangulation and lessened potential bias. The obtained information was audio recorded after obtaining verbal informed consent, and detailed notes were taken during the interactions. The recordings were transcribed by the four members, translated to English, and the team leader back-translated to ensure consistency. For quality control a sample of transcripts were checked by a senior translator, who compared them with the original audio recording. Quantitative data was summarized as numbers, and summarized in a table and bar graph. On the other hand, qualitative data was manually transcribed to form concepts. The emerging concepts were categorized based on the interventions, coded, and subjected to conventional content analysis using thematic approach, with typical and atypical statements identified for illustrative purposes. Guidance statements were organized according to the interventions and the strategies used.

### Program outcomes

This KT initiative has supported the community of Isingiro District to upscale the use of ITNs. It was well received by the communities, and it is plausible that this implementation positively enhanced the uptake of ITNs.

## Ethics statement

The KT was part of research that explored knowledge, attitude, and behavioral practices towards the use of insecticide treated mosquito nets among pregnant women and children under-five years in Isingiro district. Ethical approval was obtained from the research and ethics committee of Mbarara University of Science and Technology. As this study involved community sensitization which presents no more minimal risks and written consent was difficult considering the masses involved, informed documented verbal consent was obtained from all the participants, and this was approved by the research and ethics committee of Mbarara University of Science and Technology. For community entry, authorization was obtained from the District Health Office, Isingiro district and participant confidentiality was upheld. No incentives were given, and anonymity of the respondents was ensured at all stages of data analysis.

## Results

This KT used varied interventions to upscale ITN utilization, as presented in Table 2. The dissemination activities targeted different communal mobilizations. Dissemination strategies focused on ITN utilization as a malaria prevention approach, and included: focused health talks targeting pregnant women and those with children under-five years of age, formation of women volunteer groups, formation of malaria prevention clubs, organization of VHT groups and sharing of success stories. The findings of each intervention are presented below;

**Table 2.** Summary of interventions in the KT initiative.

Intervention	Output
Communal sensitization meetings and health talks on malaria infection, its presentation and prevention	17 communal gathering, 6 religious crusades, 3 at open markets, 7 among primary and secondary schools, and 1 meeting with the district administration.
Monitoring and support through random door-to-door home visits	46 households were visited. We ensured ITN possession in all (distribution for those that did not have was done), well hanged, mended small to medium sized damages in the ITN, and emphasized correct and consistent use.
Emphasized ITN utilization as a malaria prevention strategy	This was a focus of the community sensitization. The major output was in health talks, drama plays, folksongs, question and answer sessions, and riddles.
Care for ITNs	We held a question and answer session during health talks to all communal gatherings to emphasize the care for the ITNs, and in the monitoring and support intervention, we helped to mend those ITNs that had been torn, with replacement of those damaged beyond repair.
Promoted ITN use through community based initiatives	With support of village health teams (VHTs) and local council authorities (LCs), we formed 2 women groups with focus on malaria, 3 malaria prevention clubs, and emphasized to these authorities the need to monitor ITN use. We also shared ITN use success stories.

### Communal sensitization on malaria

The team informed and emphasized to the communities that malaria is an infection transmitted by female anopheles’ mosquito, and the symptoms that a person suffering from malaria presents with were explained, as well as the available prevention strategies.

A typical sensitization message in the direct English translation was as follows:

Malaria is a disease caused by a female Anopheles mosquito when they bite and suck blood of an infected individual. Individuals infected with malaria usually feel very sick and they develop signs of high temperatures, shivering, headache, vomiting, nausea, and sweating. If discovered early, malaria is treatable and one gets cured. It is also preventable through methods like use of insecticide treated mosquito nets, use of prophylactic antimalarial drugs, indoor insecticide spraying, repellants, and proper household hygiene to avoid water collection near homesteads and bushy environment. As most of us are aware, the government of Uganda has distributed at least a mosquito net to every two members of a household, the nets are effective, if well and consistently used. It is our responsibility to prevent malaria to avoid direct costs to treatment, and time loss when we are admitted in clinics and hospitals, or we are taking care of our relatives when they suffer from malaria; remember at worst, malaria kills.

This kind of discussion raised questions for the team and the VHTs to answer. A typical question that arose as a barrier on ITN utilization was: ‘*How do you know that a person with signs of malaria such as fever, loss of appetite and vomiting among a pregnant woman is not due to pregnancy but rather malaria?’[Pregnant woman, 28 years]* To this, a VHT member clarified that:

pregnancy can present with similar signs to malaria especially in the early stages. However, when a pregnant woman is bitten by mosquitoes, she suffers from malaria and this threatens the pregnancy. Therefore, the signs are cross cutting, but our role is to ensure that we are protected from mosquito bites using ITNs, and the women take the antimalarial drug (fansidar) provided to them during the antenatal care visits. It is important to seek for early treatment at a health facility when such symptoms occur. [VHT, 38 years]

### Monitoring and support

**Figure 1.**
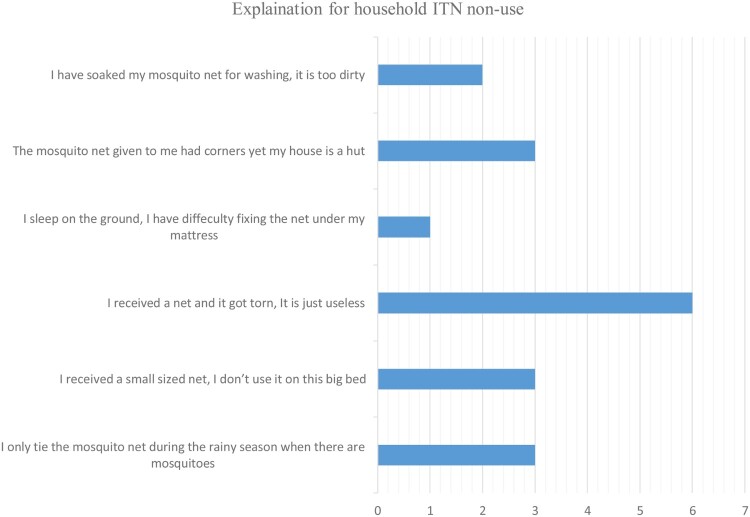
Explanations for household ITN-non use.

Of the 46 households, eighteen (17.4%) were not using ITNs properly. The findings for ITN non-use have been summarized in Figure 1.

The affected households were reminded of the positive attributes of using ITNs. Some households that required an ITN by virtue of having an at-risk member (pregnant woman or child under-five years) or the ITN was torn beyond repair, received a replacement.

### Use of ITN as a malaria prevention strategy

Clarity regarding the chemical used in the ITNs to cause cancer was made as quoted:

While there are people in our communities who have died of cancer, it is a wrong belief that the smell due to the chemical in our bed nets causes cancer. This chemical has been tested and found to repel and kill mosquitoes, but not causing cancer. This is the truth, and it has been scientifically tested. To reduce on the strong smell of the bed nets, you are advised by the VHTs to always dampen them by leaving them outside for a night, and you expose them to sunlight for a day before you hang them on a bed. [Team member, health worker]

Also, an explanation was offered to the community concern that even when ITNs are used regularly, they still suffer from malaria. In response, we highlighted that this was likely, as some people spend long hours of the night outside unprotected. Also, the team shared the different aspects of ITN use, as quoted in how ITNs work*:*

Mosquito nets act as a protective barrier against mosquitoes. In addition, ITNs can kill or disable mosquitoes by contact with the insecticide. ITNs have three main functions: (a) ITNs reduce contact between the person and mosquito by acting as a physical barrier; (b) When mosquitoes are in contact with the ITN, the insecticide in the nets kills them, and (c). The insecticide in the nets also has a *repellent effect*, that is, it prevents mosquitoes from coming close to a person sleeping under ITNs. The repellent effect adds a chemical barrier to the physical one, further reducing human–mosquito contact and increases the ITN protective effect. [VHT coordinator]

### Care for the ITNs:

This was explained using the established Ministry of Health Uganda guideline that: *ITNs have been tested and found to be effective up to 20 standard washes, and after washing, they have to be properly dried and re-hung.[VHT, 33 years]* A common finding was that ITNs were torn compromising their usefulness. To answer this, a VHT coordinator emphasized that: *Any holes in the net have to be mended to avoid mosquitoes flying through those openings*.

Also, it re-emerged that the chemical was strong and affected the users. To this, the team stressed the initial care that:

As the insecticide is highly active when the net is removed from the protective packaging, recipients are required to carefully dry the net for a short time before they hang it to prevent any possible reactions from direct skin contact. [Team member]

### Promoting ITN use:

*A life-saving story*: ‘I returned from gardening, and my 6-months baby boy had diarrhea, and had very high temperatures. At first I thought it was due to feeding him on a dirty milk bottle. I got a half of Panadol tablet, smashed it, and gave it to him, and I also prepared the oradex (oral rehydration salt) which I had received from the health center before. I gave him, later in the night at around mid-night, he became too weak so I was forced to rush him to the health center, where he was admitted. He was tested, and the medical worker asked me if I was using a mosquito net at home. I lied to her that we use it, however, on further discussion I disclosed that some nights we do not. The health worker told me that my child had a lot of malaria, and this threatens his growth. The child was treated and we were discharged. On the account of what my child went through, I shared with my husband and we agreed that we will continuously use mosquito nets. We got a relief! We have now spent close to four years, even the youngest child has never suffered from malaria. They [children] get sick but these are mainly due to diarrhea. Whenever we go for immunization, the health workers at the health center encourage us to constantly use mosquito nets, and I always testify to this. I have now lived to encourage other mothers to constantly and correctly use the mosquito nets as we were taught by the VHTs'. [Mother of three children].

*Another success story was based on the experience from a miscarriage*: during an antenatal visit to Rwekubo Health Centre IV, the team met expectant mothers, one of whom shared this narrative with us:

I am married to a man who was once a traditional healer. Three years ago, I got pregnant, and started vomiting too much, however, this was mistaken as a sign for normal pregnancy. By the time I reached the health centre, the health worker told me that I had malaria. I was admitted, put on a drip (intravenous medication) and after three days, I got a lot of pains in my lower abdomen, unfortunately I lost my baby. Most of my friends and relatives did not believe that this was malaria, but rather attributed it to my husband's cultural practices. Nurse N asked me whether I was sleeping under a mosquito net, and I told her that we did not have one. She offered me one, I went back home and shared with my husband. After like four months, I got pregnant, and delivered well. Nurse N kept closer to our family, and asked us to continuously use the mosquito net. Our baby made two years, and in all that time whenever we would go to the hospital he was never treated for malaria. [Mother of one, with a history of a stillbirth due to complicated malaria]

## Discussion

Despite many countries where malaria is endemic achieving high ITNs ownership coverage (Krezanoski, 2016; Noor, Amin, Akhwale, & Snow, 2007; Taylor, Florey, & Ye, 2017), a utilization gap exists that necessitates urgent intervention (Admasie, Zemba, & Paulos, 2018; Azeb, Habtamu, & Yitayal, 2018; Taremwa et al., 2017; Tassew, Hopkins, & Deressa, 2017; Teklemariam, Awoke, Dessie, & Weldegebreal, 2015). This study used a KT model to advance correct and consistent ITN use by improving knowledge and awareness about malaria, and encouraging the uptake of the ITN prevention strategy. It was well received by the communities and ITN use increased. This supports a previous report, where Ankomah *et al.* noted that use of health education to promote ITNs use among pregnant women in Nigeria led to an increase in their utilization ITN usage (Ankomah et al., 2014). The KT strategy offers great benefits as it often engages community members who later become innovators in disseminating the information; thus increasing the uptake of interventions. Based on this, it is plausible that this intervention in Isingiro district will continue to positively enhance and promote uptake and utilization of ITNs. Continuity requires a consolidated approach in which stakeholders encourage KT, so that interventions are based on grounded evidence. This correlates to previous reports, as the basis for KT support (Lane & Rogers, 2011; Moat, Lavis, Clancy, El-Jardali, & Pantoja, 2014; Ongolo-Zogo, Lavis, Goran, & Sewankambo, 2018). It is irrefutable that researchers ought to engage with communities to communicate their findings for purposes of influencing public opinion and advocate for policy change. Research learnings that are only published but not made known to the community defeat the goal of the research to improve health outcomes. Others have similarly commented on this (Moat et al., 2014; Nabyonga Orem et al., 2013), a practice that necessitates urgent and consistent consideration of KT implementation.

The model used in Isingiro district varied methodical approaches based on ‘edutainment’ to increase ITN uptake. Several of the strategies used have been utilized in other settings. In Nigeria and sub Saharan Africa, mass media was used to sensitize pregnant women on the benefits of consistent ITN use (Ankomah et al., 2014; Yaya, Uthman, Amouzou, & Bishwajit, 2018). Further, in Gambia, educative songs performed by community members were found to be effective in encouraging people to repair bed nets (Panter-Brick, Clarke, Lomas, Pinder, & Lindsay, 2006). The print media (brochures) and community drama have been widely used in behavioral change communications (BCC), and they have proven key for health behavior modification (Black et al., 2017; Perry, Rassekh, Gupta, Wilhelm, & Freeman, 2017; Rahman, Leppard, Rashid, Jahan, & Nasreen, 2016). The use of an edutainment model supports earlier interventions, and in all, edutainment has been proven efficient as it implores individual identities, values, beliefs and perceptions (Rentfrow, Goldberg, & Levitin, 2011; Schäfer, Sedlmeier, Städtler, & Huron, 2013). This has inexorably led to its wide utilization, a phenomenon that is in agreement with earlier reports (Fayoyin & Nieuwoudt, 2017; Malhotra, Sharma, Srinivasan, & Mathew, 2018).

Our KT intervention was limited by the following: public engagement using local frequency modulation (FM) stations, and film shows was feasible; however, it was too costly to incorporate. Our team did not directly assess community literacy before some of the health promotion messages were developed, however our previous experience from this same community (Taremwa et al., 2017) in part gave us an idea of their understanding. Accordingly, communities of Isingiro district could recognize well the signs and symptoms of malaria infection (*N *= 364, 98.6%), and knew ITN use can prevent malaria (*N *= 362, 98.1%). Despite the seemingly good understanding of these, it did not translate into consistent use of ITNs. With this in mind, our interventions were directed to address such non-compliances and suited well this community. Also, our community interventions involved the VHTs who acted as community representatives for both targeted information dissemination and also community entry. Additionally, being a cross sectional intervention, we did not elucidate the successes and acceptability of our interventions. Relatedly, this intervention is limited by the fact that our team did not integrate future planning and review meetings, and it is difficult to determine the endurance of our intervention in these communities. We recommend that future KT initiatives integrate them. This KT model was able to only incorporate 46 home visits to physically check the possession, and utility of ITNs. Thus, we are not sure if all the households have and use ITNs. Also, this KT was limited to a few parishes in one district in Uganda, hence the other districts burdened with malaria may not have a chance to benefit from this venture despite plausible benefits. It would be interesting to determine if the VHTs involved further shred their learnings, the stories and the strategies with other VHTs outside of their villages, i.e. determine if there was a ripple out effect.

## Conclusion

The notion of KT has been widely endorsed, and is premised on the potential to bridge the gap between researchers, communities, and policymakers. This undertaking describes a consolidated community intervention to promote ITN utilization. It is plausible that this intervention positively enhances and promotes uptake and utilization of ITNs.

Although a vital component, its enactment remains a challenge, mostly ending only as a proposed aspect. Many researchers attribute this to the limited operational, or no-funds at all, and this jeopardizes the research-to-implementation pathway. MicroResearch, our funder has specific KT grants for those who have completed and published their MicroResearch project. In this context, our team contributed to the utilization of ITNs as a strategy to circumvent malaria burden in Isingiro District. To the best of our understanding, this is the first KT that has been implemented in this district, and it describes an approach for community involvement to improve ITN utilization through a consolidated involvement of VHTs, local council and religious leaders. The incorporation of the innovative KT model in Isingiro district was feasible. We highly recommend integration of KT in research activities to disseminate and improve the practices of communities, and improve their healthcare standards.

## Availability of data and materials

We did not obtain consent to share data obtained from the field, however the datasets used and/or analyzed during the current study available from the corresponding author on reasonable request.

## Abbreviations

Abbreviations: BCC: Behavioral Change Communications; IEC: Information Education and Communication; ITN: Insecticide Treated Mosquito Net; KT: Knowledge Translation; VHT: Village Health Team
